# A clinical trial to compare the effects of aerobic training and resistance training on sleep quality and quality of life in older adults with sleep disturbance

**DOI:** 10.5935/1984-0063.20220040

**Published:** 2022

**Authors:** Shailly Gupta, Kshitija Bansal, Prerna Saxena

**Affiliations:** Amarjyoti Institute of Physiotherapy, Physiotherapy - Delhi - India.

**Keywords:** Resistance Training, Quality of Life, Sleep Deprivation, Aerobic Treatment

## Abstract

**Objectives:**

To compare the effects of aerobic and resistance training on sleep quality and Quality of Life (QoL) in older adults with sleep disturbance.

**Material and Methods:**

An experimental study was conducted on 36 subjects with sleep disturbance of age ≥60 years. The subjects were assessed for sleep quality and quality of life by using Pittsburgh sleep quality index (PSQI) and WHOQOL-BREF questionnaires. Those who scored PSQI>5 were included in the study and were divided into two groups A and B. Both the groups A and B received warm up and cool down with, aerobic training for group A and resistance training for group B, respectively. The training was given for 40-45 minutes a day, 4 days/week for 4 weeks. Base line and post treatment PSQI and WHOQOL-BREF scores were recorded.

**Results:**

Repeated measure ANOVA with Post hoc analysis was done using SPSS software version 16.0. Analysis revealed significant improvement (*p*<0.05) in PSQI scores within both groups but not between the groups. Post hoc analysis for sleep efficiency, sleep onset latency and sleep duration showed better improvement in resistance training group. Further QoL showed no significant difference between groups for all domains except for domain 3, i.e., social relationship which was found to be better in aerobic training group.

**Conclusion:**

Resistance and aerobic training improves both quality of sleep and quality of life. But resistance training has better effect on sleep efficiency, sleep onset latency and sleep duration. As both the training has beneficial effects on sleep quality and quality of life. So, either form of training can be used according to the individuals’ functional and medical status to improve sleep quality.

## INTRODUCTION

Sleep is the state of resting in which body is inactive and mind is unconscious. Normal healthy sleep is characterized by sufficient duration, good quality, appropriate timing and regularity, and the absence of sleep disorders. The National Sleep Foundation, 2015 recommends that older adults (>65 years) need about 7 to 8 hours of sleep each night^1^. Sleep plays a critical role in brain function and systemic physiology and is required for good physical health, mental health, quality of life, etc.^2-4^. Thus, quality of sleep is an important parameter of health. Sleep disturbances refers to the disturbance in the sleep cycle and the quality of sleep. Sleep problems include difficulty in sleep maintenance, decreased sleep efficiency, difficulty in sleep initiation, early morning awakenings, insomnia, etc.^5-7^. Along with these problems, there is less time spent in the deeper stages of sleep and also there is less total sleep time^8^. Prevalence rates of insomnia in people aged 65 and over ranges between 12 and 30%^9^. The 2003 National Sleep Foundation survey showed that one third of older adults aged 64 years and above had at least one sleep related complaints such as difficulty in falling asleep, being awake a lot during the night and getting up too early in the morning^10^. Sleep disturbance is common in older age group because of many reasons such as physical illness, changes in activity, psychological status, sleep disorders, etc. It can lead to deleterious short-term and long-term effects on the body^7^. Raised stress responsivity; decrease in the Quality of Life; mood disorders, mental health problems; memory, etc., are some of the short-term consequences while long term consequences include - hypertension, dyslipidaemia, cardiovascular disease, etc.^7^. Literature suggests that sleep disruption can lead to the increase in the risk of certain cancers and death^7^, with sleep disturbance, being a major health problem it needs assessment and rehabilitation. Though it is an important assessment parameter especially in elders, it lacks identification in the clinics^11^.

The treatment approaches for sleep disturbances includes pharmacological as well as non-pharmacological. The primary pharmacological management of sleep dysfunction are sedative hypnotic medications such as benzodiazepines, zolpidem^9,12^. The non-pharmacological interventions include cognitive behavioural therapy (CBT), physical activities that includes aerobic training, yoga, resistance training, stretching, etc. Among these two treatment options, most commonly adopted option for improving sleep problems in today scenario is the pharmacological management. Due to the lack of awareness about non-pharmacological management for sleep disturbance among the individuals, they rely on medications which offer shortterm but immediate effects. Though it is a treatment of choice, it raises major concerns like tolerance and dependency, adverse side effects, withdrawal effects, long-term health risk, financial burden due to high cost and can be associated with mortality^13^. More over non-pharmacological treatments like exercises do not have such side effects rather it shows additional benefits on health. This makes it more efficient treatment option.

Recent literature also suggests that a significant improvement in sleep quality has been observed in individuals undergoing non-pharmacological treatments. Studies also compared these treatment options like sleep diary, cognitive behavioural therapy (CBT) and physical activity like aerobic exercises, resistance exercises, etc.^14,15^. Aerobic training, resistance training, flexibility exercises has been studied for its effect on sleep as separate exercise regimes. However, there is paucity of literature about comparison between different types of exercise regimes for treating individuals with sleep disturbances. As aerobic training and resistance, training has its own beneficial effects on improving the health and also sleep when compared to other non-pharmacological treatment options. But, there is dearth of literature suggesting which out of the two trainings offer better results. So, it is necessary to find the better form of exercise which will be cost effective and efficient in improving various sleep parameters. Thereby, defining a need to set a targeted treatment approach, which can be practised clinically and improves sleep and overall Quality of Life. Though the high prevalence rate of sleep disturbance in older adults only 15% of patients report to the physiotherapy department for treatment^16^. It might be due to the lack of knowledge regarding sleep disorders and the treatment options available. Our aim will also create awareness regarding physiotherapy treatment for sleep disturbance in older adults.

## MATERIAL AND METHODS

### Participants

An experimental study was conducted on 36 older adults (18 participants in each group) of both genders who had sleep disturbance. The inclusion criteria were: age >60 yrs, PSQI score >5, individuals who reported that they were independent in their daily activities and can understand spoken English. While individuals with WOMAC score >34 in function subscale and had any acute medical illness, any diagnosed neurological conditions, severe cardio-respiratory condition, any recent fractures of upper and lower limb, replacement and any other surgery in last 6 weeks, uncontrolled diabetes, hypertension were excluded from the study. The subjects were recruited from outpatient department (OPD) of Amarjyoti Institute of Physiotherapy and Residential Welfare Associations (RWA’s) of Delhi. The data was collected from 1^st^ August 2019 to 1^st^ February 2020.

### Consent

Prior to participation, all research procedures were explained to participants in detail and written informed consent was obtained. The ethical clearance was received from Amar Jyoti Institutional Review Board (AJ-IRB). This study was registered with the Clinical Trial Registry-India, number: CTRI/2019/08/020773.

### Procedure

A screening questionnaire was developed by the researcher. The questionnaire included the questions whether the subjects were suffering from hypertension, diabetes, loss of balance and fracture in last 6 weeks, any neurological, cardiorespiratory conditions and had undergone any replacement surgery. The subjects were screened by this questionnaire and those who met the inclusion criteria were informed about the procedure of the study in detail. Convenience sampling was adopted in this study. After receiving the informed consent form, their pre intervention scores for PSQI and WHOQOLBREF questionnaires were recorded and then the subjects were randomly allocated into 2 groups using lucky draw technique. Group A received the aerobic training while group B received the resistance training. The researcher gave the sessions of both the training. Post scores for WHOQOL- BREF and PSQI were recorded by the researcher.

### Interventions

**Figure 1:**
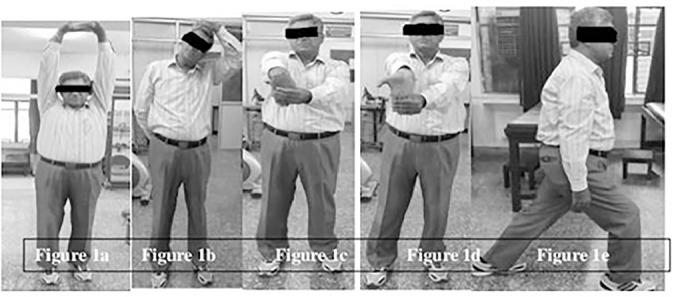
Warm up exercises: **a.** Whole body stretch; **b.** Upper trapezius stretching; **c.** Wrist extensor stretch; **d.** Wrist flexor stretch; **e.** Lunges.

Sessions of both the groups included 5 to 7 minutes of active warm up period followed by 30 minutes of respective training protocol and was concluded with 5 minutes of cool down period. The training was given for 4 days in a week for 4 weeks. Active warm up for both groups included whole body stretch, upper trapezius muscle stretch, wrist extensor stretch, wrist flexor stretch and lunges (Figure 1). All these warm up exercises were done for 3 times with hold time duration of 15 seconds.

Cool down period of 5 minutes for both groups included Shavasana; where the subjects were asked to lie in supine position with eyes closed and relax the body^17^.

**Figure 2:**
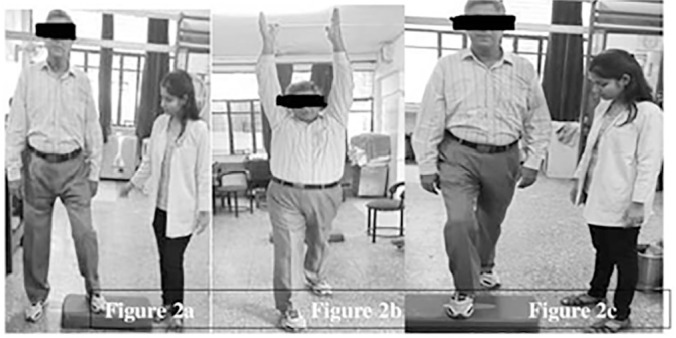
Aerobic training: **a.** Side step ups; **b.** Walking with clapping; c. Front step ups.

**Aerobic training protocol:** each session included 15 minutes of walking at point of 5 to 6 (moderate intensity) in a scale of 10 of rate of perceived exertion and 15 minutes of general body exercises which comprises of side stepping to the right and to the left 4 times in each direction, 3 sets of walking 3 steps forward with clapping, and walking 3 steps backward with clapping and four sets of stepping forwards (Figure 2).

**Figure 3:**
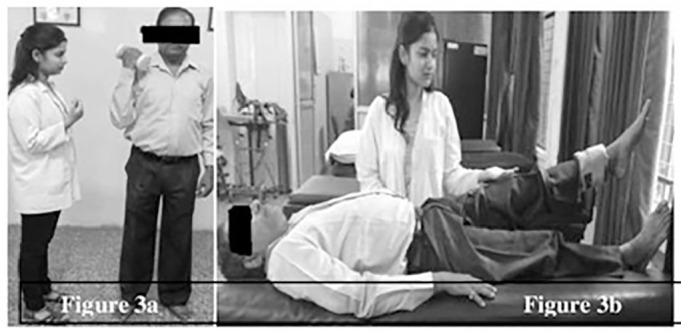
Resistance training: **a.** Biceps; **b.** Hip flexors.

**Resistance training protocol:** each session included 6 exercises for upper limbs: biceps, triceps, pectorals, shoulders flexors, extensors and abductors; 5 exercises for lower limbs: hip flexors, extensors, abductors and knee flexors & extensors. These exercises were performed at 50% of 1 RM with 1 set of 10 repetitions. Resistance exercise for biceps and hip flexors are shown in Figure 3.

### Outcome measures

#### Pittsburgh sleep quality index (PSQI)

PSQI is a self-reported questionnaire of 19-items that evaluates the quality of sleep over the past month. It distinguishes poor from good sleep quality by measuring 7 areas: total sleep duration, any disturbance in sleep, latency in sleep onset, sleep efficiency, usage of sleep medicine, daytime sleep dysfunction, and overall quality of sleep. A global score >5 indicates poor sleep quality. It is a reliable and valid scale used to assess quality of sleep. Its ICC value is r=0.71^18^.

#### The World Health Organization Quality of Life (WHOQOL)-BREF

It is a questionnaire that can be self-administered and evaluates the quality of life over the past month. It consists of a total of 26 questions which measures the following domains: domain 1 - physical health; domain 2 - psychological health; domain 3 - social relationships; and domain 4 - environment. The scoring is done by calculating the raw score of each domain and then converting the raw scores into transformed scores with the help of manual of the questionnaire. The raw score of each domain is calculated by adding the individual scores of all the questions of each domain^19^. The test-retest reliability was reported to be 0.95, which is highly significant^20^.

### Statistical analysis

Data was analysed on descriptive statistics of SPSS version 16.0. The demographics of the subjects were analysed by descriptive statistics. Repeated measure ANOVA was used to determine the improvements in pre and post PSQI and WHOQOL-BREF scores, within and in between both the training groups. In addition, Tukey’s post hoc analysis was applied to find improvement in between the group for following parameters, i.e., sleep duration, sleep onset duration and sleep efficiency, respectively.

## RESULTS

### Demographic data

**Figure 4:**
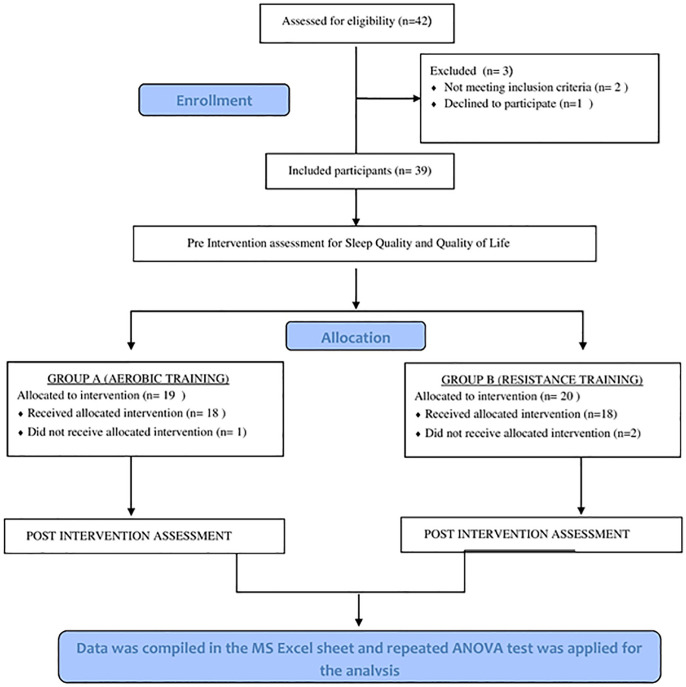
Study flowchart.

A total of 36 older adults (12 males and 24 females) having sleep disturbance were recruited (refer Figure 4). The demographic details of subjects for both groups are given in Table 1.

**Table 1:** Demographics.

Group	Gender (n=36)		Age (in yrs.)		BMI (kg/m2)		PSQI scores Mean ± S.D.	
	Males	Females	Mean ± S.D.	Range	Mean ± S.D.	Range	Pre	Post
**Aerobic**	5	13	66.5±4.98	60-75	27.28±3.16	21.5-32.9	9.05±3.18	5.33±2.40
**Resistance**	7	11	68.55±8.16	60-86	26.75±4.54	18.9-38.8	10.11±2.47	5.61±2.42

Notes: S.D. = Standard deviation; BMI = Body mass index; PSQI = Pittsburgh sleep quality index.

### PSQI scores comparison for both groups

**Figure 5:**
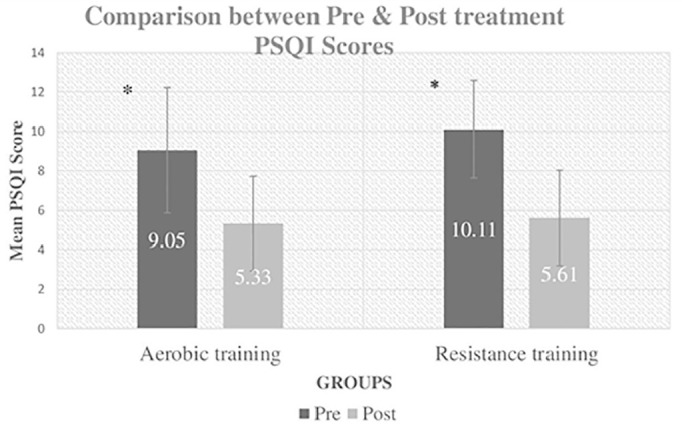
Comparison between pre and post mean PSQI scores of both groups. Notes: Main effect for time, *p*<0.05; PSQI = Pittsburgh Sleep Quality Index.

The mean PSQI scores of pre and post intervention for both groups are shown in Figure 5.

The repeated measure ANOVA analysis revealed nonsignificant main effect for group, F(1, 34) = 0.725, *p*=0.40 and a significant main effect for time, F(1, 34) = 103.378, *p*=0.000 and non-significant group X time interaction, F(1, 34) = .925, *p*=0.343, as shown in Table 2 and 3.

**Table 2:** Showing repeated measure ANOVA within the groups for PSQI scores.

Source	Time	Type III sum of squares	df	Mean square	F	Sig.
**Time**	Linear	304.222	1	304.222	103.378	.000*
Time* GROUP	Linear	2.722	1	2.722	.925	.343
**Error(time)**	Linear	100.056	34	2.943		

Notes:

* *p*<0.05; df = Degree of freedom; Sig. = Significance.

**Table 3:** Showing repeated measure ANOVA between the groups for PSQI scores.

Source	Type III sum of squares	df	Mean square	F	Sig.
**Intercept**	4080.056	1	4080.056	369.980	.000*
**GROUP**	8.000	1	8.000	.725	.400
**Error**	374.944	34	11.028		

Notes:

* *p*<0.05; df = Degree of freedom; Sig. = Significance.

#### Sleep efficiency percentage

The pre and post mean of sleep efficiency percentage for group A were 78.15±14.15 and 84.11±11.85, respectively. Further, pre and post mean scores of group B were 68.02±17.17 and 85.46±14.8, respectively. The repeated measure ANOVA analysis revealed non-significant main effect for group, F(1, 34) = 0.900, *p*=0.349. A significant main effect for time, F(1, 34) = 58.076, *p*=0.000 and group X time interaction, F(1, 34) = 14.00, *p*=0.001. Post hoc performed based on MSD = 5.79 revealed a greater improvement in resistance group compared to the aerobic group in sleep efficiency percentages.

#### Sleep onset duration

The pre and post mean of sleep onset duration for group A were 43.88±27.78 and 27.5±17.84, respectively. Further, pre and post mean scores for change of sleep onset duration of group B were 50.83±32.59 and 20±15.24, respectively. The repeated measure ANOVA analysis revealed non-significant main effect for group, F(1, 34) = 0.01, *p*=0.970. A significant main effect for time, F(1, 34 )= 44.884, *p*=0.000 and group X time interaction, F(1, 34) = 4.200, *p*=0.048.

Post hoc performed based on MSD = 13.34 revealed a greater improvement in resistance group compared to the aerobic group in sleep onset duration.

#### Sleep duration

The pre and post mean of sleep duration of group A were 5.62±1.12 and 6.22±0.97, respectively. Further, pre and post means of sleep duration of group B were 4.87±1.4 and 6.17±1.07, respectively. The repeated measure ANOVA analysis revealed non-significant main effect for group, F(1, 34) = 1.233, *p*=0.275. A significant main effect for time, F(1, 34 )= 50.670, *p*=0.000 and group X time interaction, F(1, 34) = 6.868, *p*=0.013. Post hoc performed based on MSD = 0.49 revealed a greater improvement in resistance group compared to the aerobic group in sleep duration.

#### Quality of life

**Figure 6:**
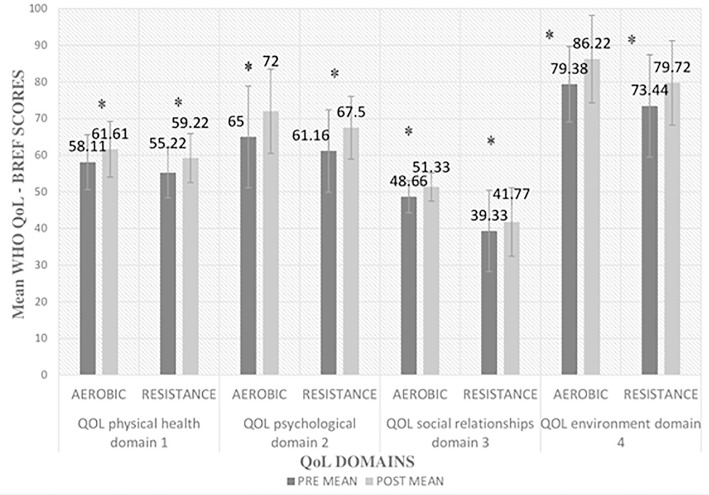
Comparison of means of Quality of life Domains of both groups. Notes: Main effect for time, *p*<0.05; QoL = Quality of Life.

The pre and post means of all domains of QoL of both the groups are given in Figure 6.

The repeated measure ANOVA analysis revealed that there is significant improvement in all 4 domains of QoL within the groups in both groups. But there is non-significant difference in pre and post scores of all domains between the groups except for the domain 3, i.e., aerobic group showed significant improvement in the scores of domain 3, i.e., social relationships as compared to resistance group.

#### Quality of life domain 1: physical health domain

The repeated measure ANOVA analysis revealed nonsignificant main effect for group, F(1, 34) = 1.671, *p*=0.205 and a significant main effect for time, F(1, 34) = 8.981, *p*=0.005 and non-significant group X time interaction, F(1, 34) = .040, *p*=0.843.

#### Quality of life domain 2: psychological domain

The repeated measure ANOVA analysis revealed nonsignificant main effect for group, F(1, 34) = 1.597, *p*=0.215 and a significant main effect for time, F(1, 34) = 12.009, *p*=0.001 and non-significant group X time interaction, F(1, 34) = .030, *p*=0.863.

#### Quality of life domain 3: social relationships domain

The repeated measure ANOVA analysis revealed significant main effect for group, F(1, 34) = 15.232, *p*=0.000 and a significant main effect for time, F(1, 34) = 6.830, *p*=0.013 and non-significant group X time interaction, F (1, 34) = .013, *p*=0.910.

#### Quality of life domain 4: environment domain

The repeated measure ANOVA analysis revealed nonsignificant main effect for group, F(1, 34) = 2.603, *p*=0.116 and a significant main effect for time, F(1, 34) = 37.250, *p*=0.000 and non-significant group X time interaction, F(1, 34) = .067, *p*=0.797.

## DISCUSSION

The results revealed significant improvement in overall sleep quality in each group. However, there was non-significant difference between the groups. Further, post hoc analysis revealed that the subjects who received resistance training had better sleep duration, sleep efficiency and sleep onset duration as compared to those received aerobic training. Moreover, there was significant improvement in overall quality of life within both groups. With aerobic training showing particularly better results in social domain of QoL. In this study, 77% of the population belongs to the age group of 60-70 years who are falling in overweight and obese (BMI>25kg/m^2^) which suggests that sleep disturbance mainly occurs in older adults, especially those who have higher BMI as a risk factor. The same fact has been reported by the Patel et al. (2008)^21^.

The study showed significant improvement in PSQI scores after 4 weeks of intervention in aerobic training group. The results of our study are also supported by various studies^10,14,22-26^. The improvement seen can be due to positive effects of aerobic excises on levels of brain derived neurotrophic factors, serotonin and norepinephrine, etc., which improves the quality of sleep. The increased level of BDNF (brain derived neurotrophic factors) helps to reverse anxiety, depression and mood swings, which is related to sleep disturbances^27^. Further, release of serotonin and norepinephrine is necessary for good quality sleep^28^. Further, it can be due to effect of aerobic exercises on cortisol levels, which are otherwise increased in sleep disorders. Definite reason cannot be suggested as the study does not include any biomarkers as outcome parameters.

We also found improvement in resistance training group. This could be due to increased energy expenditure, increased fatigue that eventually improves sleep. The subjects in this group reported mild muscle soreness and fatigue during and after exercise session. Similar results were reported by other studies^12,13,29,30^; however, according to some studies^12,13^, the mechanism is highly unclear, it becomes all the more important to study these effects with lab investigations like level of lactic acid accumulation, etc.

Both the groups were equally effective to improve quality of sleep and life except few parameters. This can be explained by effect of any form of exercise on thermoregulation, body restoration and energy conservation^9^. Rise in body temperature was observed in all the subjects irrespective to their groups. This would have activated the heat loss and improvement in sleep. As subjects were engaged in physical activity, which increased their total energy expenditure, they required more sleep than usual to restore the energy. Moreover, some soreness experienced by the subjects supported the theory of body restoration^15,31^.

To our knowledge there were dearth of studies comparing the effects of aerobic and resistance training on sleep quality in older adults. In the study, there was nonsignificant difference observed between the groups. However, post hoc analysis revealed better improvement in terms of sleep duration, sleep efficiency and sleep onset duration seen with resistance training. Similar observations were found by Faria et al. (2009)^32^, this difference can be due to the effect of session timing during day. Aerobic exercises have better effect in the evening due to reported raised melatonin levels leading to the increase in propensity to sleep^33^.

The current study did not take this factor into consideration and all the sessions were carried out in morning irrespective of their groups. Further, such effect of timing over the day has not been reported for resisted exercises. Moreover the subjects in this study reported more fatigue following resistance training as they were supposed to lift weights repeatedly that is tiring and require efforts. These two factors could have led to more sleep improvement in resistance training group.

In the study, we found improvements in all the domains of WHO QoL-BREF in both the groups, which is similar to the results obtained by some studies^14,34^ indicating resistance and aerobic training led to improvement in QoL. In this study, social relationship domain showed better results in aerobic training group. Quality of life is a parameter that declines over long standing problems and therefore can only improve over time. As this study included four weeks of intervention, which is a relatively shorter period of time to demonstrate definite changes in quality of life of older adults. However, the improvement in social relationship domain could be due to the response of group training provided to the subjects, as group training of 45 minutes have led to more social interactions between them. This in turn could have contributed to more improvement in this domain. The follow-up after few months of continuation of these training programs could have yielded better results.

### Limitations and future recommendations

Some studies suggested the effect of timing of exercises on body, which has not been taken into consideration in this study^28,35,36^; so timing of exercise should be taken into consideration in future studies. The samples in this study were not homogenous, i.e., subgroups of older adults have not been considered. In the future studies, long-term follow-up should be done with continuation of exercises at home to determine the long-term effect of exercises on sleep and QoL of older adults that was lacking in the current study. Moreover, biomarkers testing procedures to check the levels of lactic acid, growth hormone, cortisol or other brain derived neurotrophic factors should be done to have better explanation for the results.

## CONCLUSION

The study concluded that aerobic training and resistance training both are effective in improving not only sleep quality but also QoL in elderly population suffering with sleep disturbance. However, resistance training group showed better improvement in sleep duration, sleep onset duration and sleep efficiency as compared to aerobic training.

### Implications

By conducting this study, we could prove that both the training has beneficial effect on sleep quality and quality of life. Thus, either form of training can be used according to the individual’s functional and medical status to improve sleep quality. Exercise in general is beneficial for improving the sleep quality. The alternate ways of performing exercise for improving the sleep quality in older adults can vary from basic group exercises at park, yoga, stretching exercises, calisthenics, cognitive training to hi-tech solutions like exergame^5,37-40^. This study is addition to the existing knowledge of role of physiotherapy in managing sleep disorders. Further, this study will help the health care professionals especially working with sleep disorder patients to formulate an effective strategy to manage this neglected but most prevalent condition in older adults.
